# Feasibility and Perception of a Diet and Exercise Intervention Delivered via Telehealth to Firefighters

**DOI:** 10.5195/ijt.2022.6458

**Published:** 2022-06-03

**Authors:** Stephanie Donahue, Carly McMorrow, Andrew A. Almeida, Deborah L. Feairheller

**Affiliations:** Department of Kinesiology, College of Health & Human Services, University of New Hampshire, Durham, New Hampshire, USA

**Keywords:** Feasibility, Firefighters, Intervention, Telehealth

## Abstract

**Introduction:**

Firefighters have a high risk of cardiovascular incidence due to their poor health, fitness, and dietary habits. The purpose of this study was to examine the feasibility of a diet and exercise intervention within firefighters delivered exclusively via telehealth to help reduce the risks of cardiovascular disease. Additionally, the firefighters' perception of their health was assessed.

**Methods:**

Fifteen firefighters participated in a six-week Mediterranean diet and a functional circuit exercise intervention with pre- and post-fitness testing and survey completion. The firefighters had weekly video calls with their telehealth coach.

**Results:**

Self-assessed health improved with the intervention from an average of 5.9 to 7.9 out of 10. Both weight and BMI significantly decreased with the intervention. Overall, firefighters had high adherence to both portions of the intervention.

**Discussion:**

Telehealth interventions may be efficacious in improving firefighter fitness levels and overall health as firefighters saw positive health and fitness improvements.

According to the American Heart Association (AHA) the prevalence of cardiovascular disease (CVD) in adults is 49.2% (Virani et al., 2021). Interestingly, numerous studies have found that risk factors for CVD are more prevalent within the firefighter population compared to civilians (Geibe et al., 2008; Martin et al., 2019; Yoo & Franke, 2009). For instance, the prevalence of overweight and obesity (76%) among firefighters was higher than that found in the general male population (71%), and a considerable proportion of firefighters use tobacco, have hypertension, and hypercholesterolemia (Yoo & Franke, 2009) which puts them at an increased risk for developing CVD (Martin et al., 2019). In fact, 47.0% of firefighter line of duty deaths (LODD) in the United States are due to cardiovascular events (Martin et al., 2019). Despite the inherently dangerous nature of the job, firefighters are losing their lives to poor cardiovascular health. Firefighters' history of increased cardiovascular risks, poor overall health, low fitness, and the fact that firefighting work involves episodes of immediate intense physical exertion in extreme heat and life-threatening situations are all factors that account for the high rate of firefighter LODD (Elliot et al., 2007).

The National Fire Protection Agency (NFPA) created a standard known as the NFPA 1583 *Standard on Health-Related Fitness Programming* which provides the minimum requirements for a health-related fitness program for the fire department (National Fire Protection Association, 2021). Although the NFPA recognizes the importance and benefits of exercise and overall healthy lifestyles, it does not require fire stations to have a fitness program; in fact, less than 30% of fire stations have a fitness program (Donovan et al., 2009). Initially, during the hiring process firefighters are put through a battery of physical fitness tests to ensure they are “fit for duty,” and as such, evidence shows that new firefighters are typically more fit and healthy as compared to experienced ones, since they were more recently assessed for adequate fitness levels (Andrews et al., 2019). These standards tend to drop off as firefighters have been in the service for several years, since most fire stations do not require annual fitness testing to remain a firefighter, which leads to declines in health and fitness (Donovan et al., 2009). Supporting this was a recent self-reported survey which noted that as high as 46% of firefighters live a sedentary lifestyle outside of work (Lovejoy et al., 2015). As exercise science clinicians, it is imperative to assist firefighters in adopting healthy lifestyle habits to reduce LODD secondary to CVD.

It is known that blood pressure control, physical activity, and a heart-healthy diet can help mitigate the risks for CVD, yet common barriers for success are low motivation and lack of time (American College of Sports Medicine, 2018; Lovejoy et al., 2015). Also, diet interventions are typically difficult to implement, but it has been suggested that self-monitoring may be an effective tool (Gibson & Sainsbury, 2017). Based on a recent survey from Yang et al. (2015) firefighters may be likely to adopt a Mediterranean diet since it was ranked as their favorite, compared to other healthy diets (Yang et al., 2015). Recently we found that firefighters need help with adopting the Mediterranean diet (Almeida et al., 2021). A main challenge for firefighters in adopting a healthy lifestyle is motivation, so it is thought that if firefighters meet weekly with a coach, they may be more likely to adopt this healthy diet.

Telehealth can be defined as the use of electronic technologies for communication and information to provide and support healthcare when it is more feasible to reach the participant virtually and can refer to the broad range of health-related services such as patient care, education, intervention, and remote monitoring (Joo-Young et al., 2014). In fact, telehealth recently has been shown to improve exercise and diet adherence within clinical populations (Galloway et al., 2019; Kelly et al., 2019). To the best of our knowledge, a completely virtual exercise and diet study in firefighters has not been published. Therefore, the purpose of this study was to determine the feasibility, perception, and effectiveness of a telehealth exercise and diet intervention in firefighters from the perspective of both the research team and firefighters. We examined the feasibility of delivering an exercise and diet intervention by telehealth. Feasibility was measured by assessing how telehealth delivery influenced retention and adherence to the program as well as how usable the telehealth platform was. We also examined how the firefighters perceived telehealth affected their adherence to the exercise and diet components of the program.

## METHODS

### PARTICIPANTS

We used a mixed methods study design whereby qualitative data on the firefighter experience were embedded within quantitative data relating to the virtual exercise and diet intervention. Firefighters were recruited from the Dover, New Hampshire area. Overall, 24 firefighters were enrolled, and 15 firefighters completed the study. Inclusion criteria were no more than one blood pressure medication and no more than one cholesterol medication; no prior cardiovascular incidents, diagnosed heart disease or diabetes; non-smoker, and no physical limitation that would preclude an exercise program. Each firefighter gave informed consent and completed a general health history form. The protocol was approved by the Institutional Review Board.

### PROTOCOL

Firefighters completed virtual pre-testing, a six-week virtual diet and exercise intervention, and virtual post-testing within 24-48 hours of completing the intervention. The pre- and post-testing included blood pressure, weight measurements, and a fitness test. Blood pressure monitors and scales were delivered to participating fire stations. All data and testing were self-report by use of a study website and through telehealth coaching sessions. The entire study was completed using the Zoom for Healthcare software, which is a HIPAA compliant platform that ensures confidentiality and integrity of health information (Zoom, San Jose, CA). The orientation, diet training, and all fitness testing were done virtually while the firefighters were on shift at the fire station. Post-testing was administered in the same manner as pre-testing.

Blood pressure was measured using a digital monitor (Omron, HEM-7122 model; Kyoto, Japan) following clinical at-home guidelines (Shimbo et al., 2020). Firefighters were instructed to measure blood pressure for two days in the morning and at night, and the average was reported. Separately, firefighters measured their weight in lightweight clothing without shoes using a digital scale (BC-533; Tanita Corporation, Arlington Heights, IL, USA).

#### TELEHEALTH COACHES

At the start of the intervention, each firefighter was assigned to a telehealth coach and had the same coach for the duration of the study. Each firefighter met with their respective telehealth coach once per week to discuss their progress and ask any questions related to the diet or exercise program. The topics discussed during these calls were logged on a session tracking log. Additionally, within the session tracking logs, telehealth coaches recorded the quality of the audio and video during the call. Motivation is a primary barrier identified for healthy lifestyle in firefighters, so the telehealth coaches were available to make suggestions for the firefighter to be successful.

#### FITNESS TESTING

The NFPA 1583 Standard recommends annual assessments on body composition, aerobic capacity, speed and power, muscular strength, muscular endurance, mobility, and flexibility. Therefore, a variety of fitness tests were completed at pre- and post-testing (National Fire Protection Association, 2021). All fitness testing was administered virtually with at least three telehealth coaches present on the videoconferencing platform. One telehealth coach was responsible for giving directions and explanations of each fitness test to the firefighters, while the other two telehealth coaches were responsible for observing and recording times. It was feasible to test six firefighters virtually at one time using this method. Additionally, there was one lead firefighter present at the station to act as a liaison with the telehealth coaches and firefighters participating in the fitness testing. First, firefighters completed balance testing where they stood on a single leg until failure. Total time held for both legs was combined and recorded. Next, the firefighters completed a stair climb where they continuously climbed for two-minutes. Stair climb is a quality measure of firefighter fitness and is a component of the candidate physical ability test (CPAT) (Sheaff et al., 2010). The next test completed was the prone plank, which is a measure of muscular endurance used previously to test firefighter fitness (National Fire Protection Association, 2021). Following the prone plank, muscular endurance was tested again with the stationary wall sit. Cardiorespiratory fitness was measured using the 6-Minute Walk Test. Firefighters were asked to walk as fast as comfortably possible back and forth between two cones placed 30 meters apart for six minutes. Total distance walked was measured and recorded. Finally, firefighters completed the rescue drag test which is one of the tasks included in the CPAT test (Sheaff et al., 2010). For this, firefighters were instructed to drag a 65.9 kg Rescue Randy Training Manikin (Simulaids, Inc) around a cone that was placed 35 feet away and back. The exact same methods and language describing the fitness tests were used in the virtual post-testing session.

#### INTERVENTION

Firefighters were asked to complete three functional circuit workouts per week which were self-reported to telehealth coaches each week. The functional circuit described elsewhere (Mclaughlin et al., 2021), included six stations of occupation-specific exercises which was proven to be more beneficial to cardiovascular health.

For the dietary component, a virtual diet training session was provided. The diet education focused on types of food, the recommended number of servings per week, and serving sizes in each food group. Adherence to diet was tracked using a Mediterranean diet scoring system (MDSS) which gives greater importance (higher points) to foods that should be consumed more frequently. We have recently validated the MDSS which has a 17-point possible maximum, where a higher score denotes greater adherence (Reeve et al., 2021). The Mediterranean diet scoring system recommends: four or more servings of vegetables per day, three or more servings of fruit per day, two to three servings of low/non-fat dairy per day, minimize regular dairy intake, four or more servings of fish per week, less than three servings of poultry per week, minimize intake of red meat, three or more servings of beans per week, five or more servings of nuts and healthy oils per week, less than four servings of sweets and processed foods per week, and six to seven servings of grains/potatoes per day.

#### FEASIBILITY QUESTIONS

A series of questions to assess feasibility and motivation were included in the initial health history form. Follow-up questions were verbally asked to the firefighters on the final telehealth Zoom call, which was an individual call, and their answers were documented by the telehealth coach into the session log. These questions helped gauge the perception of how effective telehealth was in conducting an exercise and diet intervention. Feasibility, motivation, adherence, and perception of the intervention were quantified using a 5-point Likert scale from the answers: scores ranged from 1 being “Strongly disagree” to 5 being “Strongly agree.” Finally, a subjective rating of usability of the telehealth platform was recorded weekly by the telehealth coach in the session tracking log.

#### STATISTICAL ANALYSIS

Quantitative data were analyzed using descriptive statistics. The mean and standard deviation (SD) were calculated for quantitative variables. Data from feedback survey questionnaires were extracted and analyzed thematically. The paired *t*-tests were used to compare perception scores of the firefighters. All tests presented were two-sided and a *p* < 0.05 was considered significant. Analysis was performed using Microsoft Excel.

## RESULTS

Twenty-four firefighters were initially recruited and enrolled in the study, and 15 (14 M, 1 F, 36.3 ± 9.6 years) firefighters completed the intervention. Nine firefighters did not complete the study due to lack of time or illness unrelated to the intervention. No firefighter withdrew from the study due to issues with the telehealth delivery of exercise and diet intervention. The reported average number of years in the fire service was 13.7 ± 8.8 years.

### HEALTH AND FITNESS DATA

Table 1 presents data measures before and after the intervention. Firefighters self-assessed health improved with the intervention from 5.9 ± 1.0 to 7.9 ± 1.0 out of 10 (*P* < 0.05). Also, both weight (from 91.9 ± 12.7 to 89.1 ± 11.4 kg) and BMI (from 29.9 ± 3.2 to 29.0 ± 2.7 kg·m^−2^) decreased with the intervention (*P* < 0.05). There was no improvement in blood pressure with the intervention. Overall, firefighters had a 76.2% adherence to the Mediterranean diet and a 61.9% adherence to the exercise portion of the intervention. We also found improvements in VO_2peak_ (from 29.6 ± 3.9 to 31.7 ± 4.7 mg·kg^−1^ min^−1^), balance (from 6.4 ± 3.4 to 7.5 ± 4.3 min), stair climb (from 293.6 ± 19.1 to 335.3 ± 23.6 total steps), and muscular endurance (from 4.6 ± 1.7 to 6.0 ± 2.4 min) (*P* < 0.05 for all).

**Table 1:** Firefighter Physiological Characteristics

Variable (n = 15)	Pre	Post
General characteristics		
Age (years)	36.3 (9.6)	-
Male/Female	14/1	-
Family History (yes/no)	6/9	-
# Years as a firefighter	13.7 (8.8)	-
# Dispatch responses/mth	18.3 (9.7)	-
Health Rating (0-10)	5.9 (1.0)	7.9 (1.0) ^*^
Weight (kg)	91.9 (12.7)	89.1 (11.4) ^*^
BMI (kg · m^−2^)	29.9 (3.2)	29.0 (2.7) ^*^
Blood pressure measures		
Brachial SBP (mm Hg)	128.7 (9.4)	122.4 (11.4)
Brachial DBP (mm Hg)	77.6 (7.0)	77.7 (6.6)
Mediterranean Diet		
Adherence to Diet (%)	-	76.2 (2.8)
Average Weekly MDS	-	12.1 (1.7)
Overall Total MDS	-	70.0 (10.3)
Exercise Responses		
Exercise Adherence (%)	-	61.9 (26.7)
VO_2peak_ (mg·kg^−1^·min^−1^)	29.6 (3.9)	31.7 (4.7) ^*^
Balance (min)	6.4 (3.4)	7.5 (4.3) ^*^
Stair Climb (# steps)	293.6 (19.1)	335.3 (23.6) ^*^
Muscular Endurance (min)	4.6 (1.7)	6.0 (2.4) ^*^
Rescue Drag (sec)	17.0 (3.0)	17.0 (3.1)

* P < 0.05 with intervention. Family history, reports family history of CVD; BMI, body mass index; SBP, systolic blood pressure; DBP, diastolic blood pressure; MDS, Mediterranean diet score; balance reported as right and left leg combined; Muscular Endurance, reports wall sit + plank test times.

*Note*. Mean (± SD).

### TELEHEALTH FEEDBACK DATA

Table 2 presents the survey responses from feedback on the exercise and diet intervention via the telehealth platform. During the study, firefighters attended 82.2% (16.7% to 100%) of scheduled telehealth sessions with their telehealth coach. Telehealth sessions were missed when firefighters were responding to fire calls, were on vacation, or forgot about the appointment. It appears that firefighters “Strongly agree” (41.7%) or “Agree” (58.3%) that the telehealth sessions were useful to help them adhere to the diet. Additionally, 91.7% of the firefighters “Strongly agree” or “Agree” that telehealth sessions were useful to help change their diet. Overall, telehealth sessions were enjoyable and useful in helping firefighters feel healthier.

**Table 2:** Telehealth Intervention Feedback

	n (%)
**Sessions attended (90 total sessions)**	74 (82.2)
**Telehealth feedback from 12 firefighters:**	
**Sessions were useful to help me adhere to the diet.**	
Strongly Agree	5 (41.7)
Agree	7 (58.3)
Neutral	0 (0)
Disagree	0 (0)
Strongly Disagree	0 (0)
**Sessions were useful to help me change my diet.**	
Strongly Agree	5 (41.7)
Agree	6 (50)
Neutral	1 (8.3)
Disagree	0 (0)
Strongly Disagree	0 (0)
**Sessions were useful to help me feel healthier overall.**	
Strongly Agree	5 (41.7)
Agree	6 (50)
Neutral	1 (8.3)
Disagree	0 (0)
Strongly Disagree	0 (0)
**Sessions were enjoyable.**	
Strongly Agree	5 (41.7)
Agree	6 (50)
Neutral	1 (8.3)
Disagree	0 (0)
Strongly Disagree	0 (0)

Telehealth usability for firefighters is reported in Table 3. Video and audio quality during these sessions were excellent for 63 (85.1%) sessions, acceptable for 10 (13.5%) sessions, and poor for one (1.4%) session. Telehealth coaches conducted the sessions using a laptop for 73 (98.6%) sessions and a cell phone for one (1.4%) session. Firefighters participated in telehealth using Zoom on their cell phone for 74 (59.6%) sessions, on a laptop for 27 (36.5%) sessions, and on a portable tablet for three (4%) sessions. Additionally, 91.6% of the firefighters feel confident that they can administer a fitness test in their fire house.

**Table 3:** Telehealth Usability for Firefighters

	n (%)
Quality of audio/video during call (n = 74 total sessions)	
Excellent	63 (85.1)
Acceptable	10 (13.5)
Poor	1 (1.4)
I feel confident in administering a fitness test (n = 12)	
Strongly Agree	4 (33.3)
Agree	7 (58.3)
Neutral	1 (8.3)
Disagree	0 (0)
Strongly Disagree	0 (0)

The feasibility of telehealth was demonstrated by successful recruitment, retention, and high adherence rates of firefighters in our study. In fact, it seems that telehealth sessions were feasible and enjoyable in our study. Firefighters had positive feedback and commented:

*“I feel that my diet has improved so much because I had to report how I did each week to my telehealth coach.”*

*“I didn't realize how easy it was to make small adjustments to my diet in order to eat more fruits and vegetables instead of processed food - until we talked about it.”*

### FIREFIGHTER PERCEPTION OF ADHERENCE

**Figure 1:**
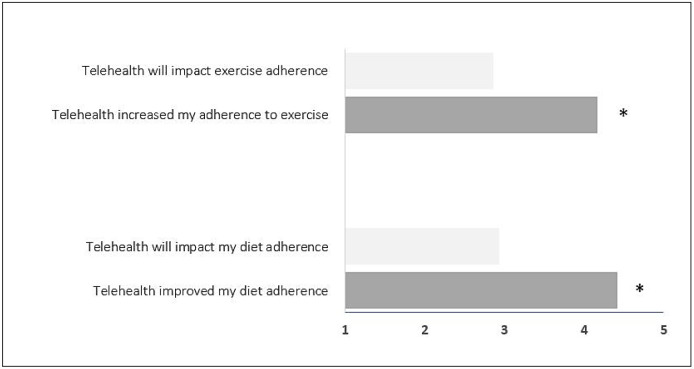
Firefighter Perception of Adherence

*Note*. Data shows mean values from perception-related pre-intervention (light grey bars) and post-intervention (dark grey bars) survey results. Firefighters (1) Strongly Disagree, (2) Disagree, (3) Neutral, (4) Agree, and (5) Strongly Agree to the statements.

Figure 1 reports perception of adherence. The average score for adherence to exercise at pre-testing was 2.86 out of 5.0. At post-testing, results suggest that perception was favorable that telehealth increased their adherence to exercise, with an average perception score of 4.17 out of 5.0 (*P* < 0.05 with intervention). In terms of diet adherence, the average score for adherence to the Mediterranean diet with telehealth was 2.93 out of 5.0 at pre-testing. At post-testing, results also suggest that perception was favorable that sessions with their telehealth coach impacted their adherence to the Mediterranean diet, with an average perception score of 4.42 out of 5.0 (*P* < 0.05 with intervention).

## DISCUSSION

Using a mixed methods study design, we found that the use of telehealth positively influenced firefighters' retention and adherence to an exercise and diet intervention. We also found that the telehealth platform, Zoom for Healthcare, was usable for both the researchers and the firefighters. Finally, we found that firefighters perceived that telehealth affected their adherence to the exercise and diet intervention. These findings are encouraging that virtual exercise and diet interventions can be effective in the firefighter population.

In our study the firefighters had improvements in body composition and rated their overall health significantly higher after the intervention compared to pre-testing. Our study also had a high adherence rate to telehealth intervention, and firefighters perceived that the intervention helped them adhere to the intervention. Chen et al. (2020) reported similar findings in pediatric heart transplant patients who participated in exercise and nutrition intervention delivered by live-video conferencing. They found improvements in BMI in the patients after a virtual exercise and nutrition intervention (Chen et al., 2020). Chen et al. enrolled heart transplant patients into a 12 to 16-week intervention that utilized live video-supervised exercise and nutrition sessions. In addition to seeing improvements in BMI, which would be expected with an exercise and diet intervention, Chen et al. had an intervention adherence rate greater than 80%, comparable to our findings. The participants in Chen et al.'s study reported the ease of scheduling at-home sessions based on their availability allowed for greater adherence overall, and all of the participants would like to continue the virtual intervention as it was feasible. While our study was not supervised, both studies report improvements in body composition and that adhering to an exercise and diet intervention utilizing telehealth is feasible.

This adherence to telehealth sessions is similar to adherence reported by Kelly et al. (2019) who assessed the feasibility and acceptability of telehealth coaching to promote healthy eating in chronic kidney disease patients. Kelly et al. used post-intervention surveys and interviews to collate data. Compatible with our findings, they found that a tailored telehealth program with phone calls and text messages increased diet adherence more than generic text messages (Kelly et al., 2019).

In our study, both firefighters and telehealth coaches found the telehealth platform to be usable. A study by Galloway et al. (2019) assessed the feasibility of a telehealth exercise program in patients after a stroke, but they report that their virtual platform was not as usable. They also reported that a high percentage of the calls were “poor” in quality. We hypothesize that this difference in findings could be a result of a clinical setting with an older population, and the participants may not have had experience with technology. Galloway et al. (2019) did, however, report high enjoyment with telehealth exercise sessions and concluded that the intervention was feasible. These are important findings that our study confirms. It seems that telehealth may be used to deliver rehabilitation, administer preventative diets, and exercise interventions. These findings could be built upon by implementing exercise and diet interventions with other first responders across the country.

Finally, firefighters in our study felt that telehealth increased their adherence to both exercise and diet. To the best of our knowledge, there are no other studies that have assessed perceptions of telehealth in firefighters with an exercise and diet intervention. In 2019, Olfert et al. (2019) reported positive effects of personalized mobile health diet counseling in a group of overweight patients. In their study, they measured perception with a 32-question behavior and attitude survey administered at baseline, mid-intervention, and post-intervention. Researchers also conducted interviews after the study was completed to obtain feedback from the participants. They found that a mobile health approach to healthier nutrition and lifestyle was feasible for a rural population. With continuous advancement of technology, it is important to explore perceptions of telehealth and mobile health among diverse populations using different interventions. The knowledge that firefighters perceived telehealth to be helpful in adhering to the intervention hints at the possibility of implementing wellness interventions more broadly among firehouses nationwide. This has broad implications for potentially reducing the cardiac risk and improving cardiac health in firefighters.

Our study has several limitations. First, firefighters from the local fire department were included in the study, so it is not representative of the entire firefighter population. Secondly, it was a small study population, but for a feasibility study this was an adequate sample size. In addition to this small population, only one female firefighter was included, thus the data may not be as easily extrapolated for female firefighters. However, only 8% of firefighters are female, which is a representative percentage of the female firefighter population. Also, we did not have a control group for this study. This may limit the overall effectiveness measure which was not a primary outcome as this study was designed to test the feasibility and perceptions of virtual interventions. Another limitation was that the exercise and diet adherence were self-reported by the firefighters which could affect true adherence levels. Many exercise and diet studies are conducted by self-report, so this study still provides valuable information on the feasibility and usability of the telehealth platform. Finally, the study was conducted during a 6-week period in the summer months which may have affected adherence due to vacation time. Future researchers should consider conducting the study for a longer period and during other times of the year. Further research should be done on a larger scale with firefighters nationwide to further assess the feasibility and acceptability of a virtual diet and exercise intervention delivered via telehealth. This small sample size saw positive results overall, and it would be expected to see similar results in other fire stations, or more broadly, with other first responders.

In conclusion, firefighters perceived that virtual implementation of an exercise and diet intervention affected and improved their adherence. Firefighters also saw positive improvements in health and fitness indices with the virtual intervention. Successful implementation of a virtual exercise and diet intervention could potentially be lifesaving for firefighters, as they are known to have a higher risk for CVD and LODD secondary to CVD. This could be an efficacious solution to reducing cardiac risk and an easy way to increase wellness programming for firefighters.
